# Beneficial Effects of Plant Oils Supplementation on Multiple Sclerosis: A Comprehensive Review of Clinical and Experimental Studies

**DOI:** 10.3390/nu15224827

**Published:** 2023-11-18

**Authors:** Ghanya Al-Naqeb, Aliki Kalmpourtzidou, Rachele De Giuseppe, Hellas Cena

**Affiliations:** 1 Laboratory of Dietetics and Clinical Nutrition, Department of Public Health, Experimental and Forensic Medicine, University of Pavia, 27100 Pavia, Italy; aliki.kalmpourtzidou01@universitadipavia.it (A.K.); rachele.degiuseppe@unipv.it (R.D.G.); hellas.cena@unipv.it (H.C.); 2 Department of Food Sciences and Nutrition, Faculty of Agriculture Food and Environment, University of Sana’a, Sana’a P.O. Box 1247, Yemen; 3 Clinical Nutrition Unit, General Medicine, ICS Maugeri IRCCS, 27100 Pavia, Italy

**Keywords:** multiple sclerosis, plant oils, polyunsaturated fatty acids, EAE model

## Abstract

Multiple sclerosis disease (MS) is a 38.5 chronic neurological autoimmune disease that affects the nervous system, and its incidence is increasing globally. At present, there is no cure for this disease, and with its severity and disabling variety, it is important to search for possibilities that could help to slow its progression. It is recognized that the mechanisms of MS pathology, its development and degree of activity can be affected by dietary factors. In this review, the beneficial health effects of 10 plants oils—mainly seed oils, including pomegranate seed oil, sesame oil, acer truncatum bunge seed oil, hemp seeds oil, evening primrose seed oil, coconut oil, walnut oil, essential oil from *Pterodon emarginatus* seeds, flaxseed oil and olive oil—on MS are discussed. The literature data indicate that plant oils could be effective for the treatment of MS and its related symptoms primarily through reducing inflammation, promoting remyelination, immunomodulation and inhibiting oxidative stress. Plant oils may potentially reduce MS progression. Longitudinal research including a larger sample size with a longer duration is essential to confirm the findings from the selected plant oils. Moreover, new plant oils should be studied for their potential MS benefit.

## 1. Introduction

Multiple sclerosis disease (MS) is a chronic neurological, inflammatory and progressive autoimmune disorder that affects the central nervous system (CNS). An increasing incidence rate of MS has been reported: 2.5 million people [1] have been estimated to be affected, with a prevalence rate of about 35.9 per 100,000 subjects [2]. It is therefore considered the most common progressive neurologic disease of young adults worldwide [3], and is more prevalent in females than in males [4]. Multiple sclerosis mainly affects the neurons, which are surrounded by a fatty layer known as the myelin sheath. This myelin sheath may be degraded by the demyelination process in MS, which leads to the transection of neuron axons in patches through the brain and spinal cord, leading to the death of axons and neurons [5]. Three main types of MS are known, including relapsing-remitting MS (RRMS), which has been recognized to be the most common type of MS, primary progressive MS (PPMS) and secondary progressive multiple sclerosis (SPMS) [6].

The symptoms of MS vary from limb weakness to the dysfunction of organs such as the bowel or bladder and mental changes, from diplopia to ataxia [7]. The neuropathological process associated with MS involves de- and re-myelination lesions, inflammatory alterations, gliosis and axonal degeneration, as well as elevated lymphocyte levels in the brain [1]. The episodes reflect inflammatory demethylating lesions in the brain, optic nerves and spinal cord and could result in different symptoms, including a loss of vision, weakness, numbness and bowel and bladder disturbances [6]. Common symptoms of MS patients are pain, fatigue, depression and a decrease in the ability to hold attention [8].

The pathogenesis of MS disease is complex and multifactorial; several factors were identified to play a role in the development of MS, as well as in the disease course and progression, including genetic and environmental factors [5,7]. For instance, vitamin D levels depletion, viral and microbial infection, smoking, sun exposure, obesity and dietary habits were reported to be relevant to the pathogenesis of MS [9,10]. Thus, MS pathophysiology is not well elucidated, which renders the treatment strategy of the disease unclear [11]. 

Animal models are essential in order to understand the induction and pathogenesis of this disease and to develop prevention or therapeutic strategies that delay its progression [12]. In general, mouse models are the most frequently used because of the inbred genotype of laboratory mice, their rapid breeding capacity, the ease of genetic manipulation and the availability of transgenic and knockout mice to facilitate mechanistic studies. Among the successes and acceptable models, the experimental autoimmune encephalomyelitis (EAE) is primarily used as an animal model of autoimmune inflammatory diseases of the CNS, and it resembles the symptoms and pathology of MS in multiple ways. Indeed, several clinical and pathological features of EAE show close similarity to the human MS disease [13]. Experimental autoimmune encephalomyelitis has commonly been used as a model system to study the mechanism of MS pathogenesis and to test the efficacy of potential therapeutic agents for the treatment of MS [12]. Although there are some notable variations between this model and humans in many aspects of MS disorders, a lot of research into the autoimmune and inflammatory processes has been conducted using this model [13].

To date, MS therapy has been supported with a variety of therapeutic approaches to modify the course and progression of the disease, as well as to reduce the disease symptoms, including medications, stem cell therapy, urinary catheterization, venous angioplasty, rehabilitation and psychotherapy [14]. So far, in MS treatment, the medications have mainly focused on preventing CNS inflammation [15]. At present, eight types of medications are used to reduce MS progression [16]; however, side effects have been reported [17,18]. There is a wide range of complementary treatments for MS diseases reported in the literature, including lifestyle, a medicinal approach through food and diet, exercise and stress management [19].

The mechanisms of MS pathology, its development and its degree of activity can be affected by nutrition and dietary factors [9]. Many studies have investigated the role of nutrition in the pathogenesis, etiology and treatment of MS. Healthy eating habits and a high-quality diet seemed to reduce the disability of MS patients and have a positive impact on physical and mental health [20,21]. Nutrition intervention studies have suggested that diet may be considered as a complementary treatment to control the progression of the disease [21].

Oxidative damage is involved in both RRMS and PPMS [22]. Inflammatory and oxidative stress mediators, including several cytokine indicators, have been clinically linked to the progression of MS [23]. Dietary antioxidant factors have the potential to mitigate oxidative stress and prevent chronic demyelination and axonal damage by controlling the activation of immune inflammatory cells and reducing inflammation [24]. According to the research, antioxidants such as fatty acids, vitamin D and curcumin may be involved in the control of oxidative stress [25]. Fatty acids, particularly ω-3 polyunsaturated fatty acids (PUFAs), are another antioxidant molecule associated with improvements in certain indicators associated with inflammation and/or neurodegeneration in MS patients [26]. Eicosapentaenoic acids (EPAs) and docosahexaenoic acids (DHAs) could also help lower MMP-9 levels in MS patients [27]. Some studies, however, found no benefit from ω-3 or EPAs and docosahexaenoic acids (DHAs) individually in MS investigations [28,29]. Ω-6 linoleic acid and its metabolite gamma-linolenic acid has been shown to be beneficial in MS [30].

Edible plant oils, mainly seed oils, are rich sources of essential compounds, including PUFAs, antioxidants and polyphenols. They are essential components of the human diet and a major source of edible lipids, which account for more than 75% of the total lipids consumed in the world [31]. We conducted a search for all English language articles in ScienceDirect, PubMed, Sage Journals, SCOPUS and Google Scholar on 10 plant oils—mainly seed oils, including pomegranate seed oil, sesame seed oil, acer truncatum bunge seed oil, hemp seed oil, evening primrose seed oil, coconut oil, walnut oil, essential oil from *Pterodon emarginatus* seeds, flaxseed oil and olive oil—and their beneficial effects on MS. These plant oils have been known for their health-benefitting activities. They contain essential fatty acids required for the proper development and function of the human body. Most of these plant oils are rich sources of linoleic acid, which has been reported to be effective in improving MS disease symptoms [32]. Linoleic acid was reported to have a role in the regulation of cell-mediated immunity [25,33]. In addition to these essential fatty acids, they are also a good source of many significant phytochemicals such as carotenoids, tocopherols, sterols and phenolic compounds, as well as vitamins and minerals.

Edible plant oils are major components of food systems; they are also important sources of energy in the human diet and an important economic commodity [34]. Plant oil production can lead to higher incomes, generate labor employment and reduce poverty [35]. Plant oils, mainly seeds oils, are either used for edible purposes such as cooking or are found to be used for medicinal uses. The present narrative review provides insights into the mentioned plant oils and their potential role in reducing MS progression. A total of 22 articles were reviewed, most of them published between 2013–2023, while one article was published in 2007. Human clinical trials, in vivo animal models and in vitro studies are discussed in this review. The possible mechanisms that show how these plant oils exert their effects on MS are covered; the phytochemicals and essential compounds that are suggested to be responsible for their effects are also covered in this review article. A summary of this review and new suggested research are included. 

## 2. Plants Oil and Multiple Sclerosis

### 2.1. Pomegranate Seed Oil

Pomegranate seed oil (PSO) is obtained from pomegranate (Punica granatum) seeds, with a content of around 12% and 20% of the total seed weight [36]. Pomegranate seed oil is rich in PUFAs, among which the conjugated linolenic acid content is up to 80% of the total PUFAs with a varied isomeric distribution [37]. Among these isomeric distributions, punicic acid was identified as the active compound in PSO [38]. Pomegranate seed oil also contains linoleic acid (13–20%), oleic acid (8–9%), linolenic acid (0.06–0.08%) and arachidic acid (0.68–0.90%) as well as important fatty acids such as gallic acid and ellagic acid [39]. Other important bioactive compounds including phenolic compounds, tocopherols and phytosterols are also present in PSO [40].

Pomegranate seed oil has been reported to have a protective effect against oxidative stress, increasing antioxidant activity and reducing inflammation biomarkers [41]. In addition, PSO was shown to reduce plasma interleukin-6 and tumors necrosis factor levels in high-fat-diet-induced obese mice [42]. Furthermore, PSO nanoemulsion prevented a cognitive and behavioral decline in mice induced with traumatic brain injury, reduced neuronal death and also prevented mitochondrial damage [43]. Interestingly, many biological effects including antioxidant, anti-inflammatory, anti-cancer and anti-apoptotic of PSO were related to the presence of a high amount of punicic acid and punicalagins [44].

Regarding MS, PSO was reported to improve cognitive dysfunction in MS patients. In this regard, in a single-center, randomized double-blind placebo-controlled clinical trial, Petrou et al. [45] investigated the effect of a 3-month treatment of nanoemulsion formulation of PSO, named GranaGard, on 30 MS patients in combination with their immunomodulatory MS-treatments. Two cognitive tests that are known to be sensitive in the detection of cognitive dysfunction—namely, the Expanded Disability Status Scale (EDSS) [46] and Multiple Sclerosis Functional Composite [47]—as well as cognitive tests including the Brief International Cognitive Assessment for Multiple Sclerosis (BICAMS) [48], were applied. The outcomes of this study indicated a cognitive improvement in MS patients treated with PSO nanoemulsion in all of the tested measures, with no adverse effect when compared to untreated patients. The possible effect of PSO nanoemulsion on the improvement of cognitive dysfunction in MS patients was suggested to be due to the antioxidative effects of PSO nanoemulsion and its main active ingredient, punicic acid. Punicic acid is known to achieve neurodegenerative prevention through different mechanisms [49] by reducing Reactive Oxygen Species (ROS) generation and lipids peroxidation in both in vitro models and in humans [50,51].

Again, in the EAE animal model experiment by Binyamin et al. [52], the administration of PSO in two different forms—as 10% oil/water nanoemulsion either through oral gavage or mixed with diet at 25 or 75 mL of PSO/kg of the diet—were conducted for 10 days. EAE female mice treated with PSO supplemented with diet showed a reduction in disease symptoms and burden. This effect was even greater when the PSO was administered as the nanoemulsion formulation. The administration of PSO in nanoemulsion form showed a dramatic reduction in demyelination and oxidation of lipids in the brains of the treated mice compared to the untreated mice. 

Additionally, it was clear that, while the administration of large doses of PSO with diet can reduce MS disease burden in EAE mice, PSO nanoemulsion exerted a wider effect at a much lower dose. Pomegranate seed oil nanoemulsion increased the bioavailability, and the activity of PSO as nanoemulsion has been reported to be an effective delivery system [53]. In addition, EAE mice treated with PSO nanoemulsion showed a reduction in malondialdehyde (MDA) levels, a product of lipid peroxidation, which was shown to be increased in the blood and serum of patients with MS [54]. Recent evidence indicates that oxidized lipids are neurotoxic and have pro-inflammatory properties, and lipid peroxidation products could be involved in demyelination and axonal injury in MS [55,56].

Pomegranate seed oil was also found to have a neuroprotective effect on transgenic mice mimicking a genetic prion disease [57], where nanoemulsion of PSO successfully inhibited the disease onset in treated mice compared to untreated mice. Lipid oxidation and neuronal loss were decreased in the treated mice, indicating that the PSO nanoemulsion had a strong neuroprotective effect. Pomegranate peel extract has been shown to have a beneficial effect against MS disease in EAE mice, where it was reported that the pomegranate peel extract resulted in a decrease in clinical symptoms, demyelination and axonal damage in EAE mice [58].

### 2.2. Sesame Oil

Sesame oil (SSO) is obtained from the seed of sesame (Sesamum indicum L.), with a content of 37–63% of oil. It is rich in PUFAs and counts for 82%, along with a balanced amount of linoleic and oleic acid [59]. Sesame seed oil is known for its nutritive and health promotion values; indeed, SSO consumption has been shown to reduce blood glucose levels and to have beneficial effects on lipid peroxidation and antioxidant levels in streptozotocin-induced diabetic rats [60]. In addition, SSO was reported to have anti-inflammatory effects through the reduction in proinflammatory cytokines, as well as antioxidative effects [61].

Regarding MS, it was reported that the combination therapy of interferon beta-1a with SSO induced immune modulation by increasing regulatory cytokines [62]. In a randomized controlled trial with 93 patients with MS, the control group (*n* = 39) received 30 μg/week of interferon beta-1a intramuscularly and the treated group with SSO (*n* = 54) received interferon beta-1a—that is, the same as the control group, with the addition of 0.5 mL/kg/day of oral sesame oil—for 6 months. As a result, interleukin (IL) 10 concentration, leukocyte proliferation and nitric oxide (iNOS), as well as inflammatory cytokines including IFN-γ and TNF-α, were significantly reduced in the patients treated with SSO compared to the patients treated with interferon beta-1a alone. The reduction in inflammatory cytokines and nitric oxide was suggested to be attributed to the presence of anti-inflammatory agents in SSO that could show anti-inflammatory effects [62]. Sesame oil was reported to contain different lignans, including sesaminol, sesamolin, pinoresinol and sesamin, and these lignans are the compounds responsible for the antioxidant and anti-inflammatory properties [63]. 

It was recognized that there is a relation between the severity of MS disease and the number of IFN-γ. IFN-γ is an important cytokine of cell-mediated immunity, which is mainly produced by macrophages and T cells [64]. IFN-γ has been reported to increase MS severity through leukocyte infiltration in the brain, the activation of macrophages and iNOS production [65]. Previous studies have indicated that during MS and EAE conditions, there is an enhancement in IFN-γ levels [66]. In addition, it was found that anti-IFN-γ has a positive impact on TH1-mediated autoimmune disorders [67]. In this regard, the cytokine modulatory effects of SSO on EAE female mice were reported by Javan et al. [68]; mice were injected intraperitoneally every day with SSO at 4 mL/kg/day for 20 days and control mice were injected intraperitoneally with 4 mL phosphate buffer. As a result, SSO was able to significantly reduce the disease severity in comparison with the control mice. Sesame oil induced TH2- and TH17-related immune responses and suppressed the TH1 type in EAE. In regard to the IFN-γ levels, IFN-γ were reduced significantly in the treated mice and the level of IL-10 production was increased in the EAE mice treated with SSO compared to the untreated mice. It was reported that IL-10 has a suppressive effect on EAE progression by acting through the modulation of TH1 responses and reducing IFN-γ production, which leads to a decrease in the disease severity [69].

An important aspect of the pathogenesis of EAE, with potential for therapeutic manipulation, is the role of ROS in the inflammatory process [70]. Thus, a scavenger of ROS is expected to prevent this free radical-mediated EAE. In the EAE MS model, intraperitoneally injection of SSO reduced the clinical symptoms of EAE and increased the total antioxidant capacity in the serum of EAE mice [71]. Typical brain inflammatory cell infiltration was observed in the EAE mice compared to the SSO-treated mice. Sesame oil effectively prevents MS disease progression in EAE mice, which was related to the inhibition of oxidative stress [71].

### 2.3. Acer Truncatum Bunge Seed Oil

Acer truncatum bunge oil (ATBO) is an edible oil obtained from the seeds of Acer truncatum bunge. The oil was characterized to be rich in PUFAs, which counts for about 92%, including ω-9, ω-6 and nervonic acid [72]. Nervonic acid deficiency has been associated with neurodegeneration, and supplementation with nervonic acid nutraceuticals has shown an improvement in brain development and cognition [73]. Several health benefits of ATBO have been reported; for instance, ATBO inhibited the differentiation of 3T3-L1 adipocytes cells by inhibiting fatty acids synthesis and reducing the number and the size of the cells, suggesting that ATBO might be used in obesity treatment [74]. In addition, ATBO had the ability to improve the learning and memory of aging mice by downregulating the inflammation factor at the gene expression level [75]. Moreover, there was an improvement in cognitive function in the memory of rats treated with ATBO because of essential fatty acids, including nervonic acid [72].

Multiple sclerosis has been known to cause abnormalities and neuroinflammation in the brain. Cuprizone-induced mice have been used as an animal model of demyelination and remyelination and for the examination of the neuroinflammation and oligodendrocyte dysfunction hypotheses [76]. Thus, the beneficial effect of ATBO administration on the remyelination process in a mouse model of MS induced with Cuprizone was investigated [77]. Cuprizone-induced mice were treated with a diet supplemented with 4% of ATBO for 6 weeks. The outcomes of this study indicated that the diet supplemented with ATBO to the cuprizone-induced mice reduced the demyelination that was induced by Cuprizone, indicating that ATBO is a novel therapeutic diet in demyelinating diseases. In addition, the dietary supplementation of ATBO inhibited microglia and astrocyte activation in vitro. A possible mechanism is that ATBO exerted its remyelination process, which was suggested to be due to an acceleration of the differentiation of oligodendrocyte precursor cells to mature oligodendrocytes. It was found that in Cuprizone-administered animals that undergo demyelination, an increase in oligodendrocyte precursor cells and a dramatic decrease in mature oligodendrocytes were observed in mice treated with ATBO, suggesting a blockade of oligodendrocyte precursor cells differentiation into mature oligodendrocytes [77].

### 2.4. Hemp Seed Oil and Evening Primrose Oil

Hemp seed oil (HSO) is known as a functional food [78]. It is rich in essential fatty acids, with a PUFAs content of over 80% [79]. The PUFAs most prominently presented in HSO are linoleic and α-linolenic acids, with a content of 50–70% and 15–25% of total oil, respectively [80]. The ω6/ω3-PUFA ratio in HSO is reported to be between 2:1 and 3:1, which is considered optimal for human health [81]. In addition, HSO contains alpha-linolenic acid (GLA) and stearidonic acid, which act as biological precursors for longer-chain ω-3 fatty acids [77]. A significant quantity of important antioxidants, including carotenoids, tocochromanols, chlorophyll, terpenes, phytosterols, tocopherols and polyphenols, has been reported in HSO [82]. Hemp seed oil has been used to treat various disorders for many years in traditional medicine. Several researchers have studied the health-benefit effects of HSO, including antioxidant and anti-inflammatory activities [83,84]. Evening primrose oil (EPO) is obtained from the evening primrose seed plant, with the scientific name of Oenothera biennis, which belongs to the family of panacea plants. Evening primrose oil is rich in linoleic acid and contains oleic acid and γ-linolenic acid (8–14%) [85].

MS is a chronic inflammatory and neurodegenerative disease of the brain and spinal cord, which leads to disability and functional loss due to demyelination and neuronal injury [86]. In MS, PUFAs exert immunosuppressive actions through their incorporation into the immune responses and affect cell function within the central nervous system [87]. Antioxidants can support cellular defenses in various ways, including radical scavenging, interfering with gene transcription, mRNA ex-pression, enzyme activity and chelation. Both dietary antioxidants and PUFAs have the potential to reduce MS disease symptoms by targeting specific mechanisms and supporting recovery in MS [88]. In this line, a study was reported by Rezapour-Firouzi et al. [89] that used the EAE MS animal model. Female mice were treated with a combination of EPO/HSO for 2 weeks at 50 λ/mouse orally. The percentage of essential fatty acids, including linoleic, gamma-linolenic acid, dihomo-γ-linolenic acid and arachidonic acid, and ratios of polyunsaturated fatty acids (ω3/ω6-PUFAs) significantly elevated the cell membrane of the spleen and blood of linoleic, gamma-linolenic acid, dihomo-γ-linolenic acid and arachidonic acid in blood samples of treated animals in comparison with untreated animals. In addition, the relative expression levels of IL-4, IL-5 and IL-13 genes in the lymphocytes and serum levels of IL-4 were significantly increased in the HSO/EPO-treated animals compared to the untreated animals. Moreover, the histological assessment showed no demyelination in the brain and spinal cord sections of the EPO/HSO-treated mice in comparison to the non-treated mice. The positive effect of the combination of both oils for remyelination for the treatment of EAE is suggested to be because of the antioxidants and PUFAs presented in both oils [89]. Of importance, arachidonic acid is a precursor of pro-inflammatory prostaglandin (PG)E2, but docosahexaenoic acid and di-homo-γ-linolenic acid are precursors of the anti-inflammatory PGE3 and PGE1 series [90]. Because of the effective anti-inflammatory activity of GLA, EPO is regularly recommended for the treatment of inflammatory and autoimmune disorders. The earliest results of the use of EPO and colchicine combined therapy in MS patients suggested that it may be of considerable value [91].

In the immune system, it was identified that the T regulatory cells act as suppressors of T cells, which are a subset of T cells that modulate the immune system, maintain tolerance and prevent autoimmune disease. In the EAE MS model, interleukin 10, derived from T regulatory cells and T helper, is known as an anti-inflammatory cytokine that can prevent and/or reverse EAE symptoms [92]. A previous study showed the effectiveness of rapamycin (RA-PA) as an inhibitor of mTOR signaling in the development of tolerance through the expansion of T regulatory (Treg) cells [93]. In this line, the immunomodulation and remyelination activities of a combination of HSO/EPO supplements on the EAE MS model in comparison with RAPA were investigated [92]. The findings from this study indicated that the diet supplemented with a combination of both HSO/EPO was more potent in downregulating the disease symptoms of EAE compared to RAPA. The expression level of the IL-10 gene was significantly increased in the HSO/EPO group compared to the untreated group. In contrast with RAPA groups, histological findings have shown that the HSO/EPO-treated group remarkably reduced cell infiltration and promoted remyelination. HSO/EPO could exert its effects through immunomodulation and remyelination activities, which could potentially be used in MS treatment [92].

Additionally, EPO was found to reduce overall life satisfaction in patients with MS. Majdinasab et al. [94] conducted a double-blind randomized clinical trial of 52 MS patients and categorized them into two groups, receiving 1 g oral capsule containing EPO every 12 h for 3 months of EPO or placebo, in addition to the standard treatment for their disease. The findings from this study showed that EPO consumption showed a significant effect on increasing cognitive function, vitality and overall satisfaction with life and a significant reduction in fatigue and pain in patients with MS compared to untreated patients [94].

As the liver is the main organ for drug detoxification and digestion, it was shown that the liver enzyme levels were elevated due to the treatment of MS-like Interferon-β (IFN-β) [95]. Interferon-β was shown to shift the immune response from the Th1 to Th2 pattern by enhancing the anti-inflammatory Th2 cytokines and decreasing the production of pro-inflammatory Th1 cytokines. A double-blind randomized trial with MS patients was conducted to investigate the effect of the combination of EPO/HSO on the liver enzymes activity, including alanine transaminase (ALT), aspartate-aminotransferase (AST) and gamma-glutamyl transferase (GGT) [96]. The treated patients received a combination of HSO and EPO with a 9/1 ratio at 18–21 g/day (6–7 g, three times daily) and the control patients received the same dose of olive oil. The study indicated that diets supplemented with virgin EPO/HSO for 6 months resulted in a reduction in enzyme activation compared to untreated MS patients, a reduction in clinical symptoms of MS and the patients’ general health improved. The possible mechanism suggested for this effect is due to the antioxidant compounds that are presented in both oils, which were responsible for improving the activity of liver enzyme [96].

In addition, Rezapour-Firouzi et al. [87] investigated the immunomodulatory and therapeutic effects of a combination of EPO/HSO intervention on MS patients. The findings from this double-blind randomized clinical trial with 20 MS patients in treated and control groups indicated that a 9:1 combination of HSO and EPO as a dietary supplement in a daily dose of 18–21 g/day over a period of 6 months showed clinical improvements in terms of an expanded disability status scale and relapse rate in MS patients with HPS/EPO intervention. Also, a significant reduction in the pro-inflammatory cytokines IL-17 and IFN-γ was observed in the treated group with HPS/EPO compared to control patients. The possible mechanisms of the HPS/EPO effect were suggested to be related to the PUFA present in both oils, and their metabolites affected inflammatory functions and cytokines production during the 6 months because ω3-PUFAs can suppress IFN-γ production in MS patients [87].

Moreover, Rezapour-Firouzi et al. [97] investigated the regulation of lipid-dependent membrane enzymes through a combination of both HPS/EPO interventions in MS patients. The patients who received the HPS/EPO intervention experienced a significant increase in their red blood cells PUFAs rate compared to the control patients, while a significant decrease in phospholipase-A2 level was observed. As it is known, phospholipase-A2 controls the metabolism of PUFA. Phospholipase-A2 plays a role in cell injury in the CNS, as well as in the pathogenesis of MS and the production of pro-inflammatory mediators [98]. The phospholipase-A2 hydrolyzes phospholipids to release arachidonic acid, which can mediate inflammation and demyelination, which are hallmarks of the CNS autoimmune disease MS [99]. The phospholipase-A2 concentration was found to be increased by up to 6-fold in the urine of MS patients with active disease and 4-fold in patients in remission, regardless of the immune-modulating therapy [100].

Another study by Rezapour-Firouzi et al. [101] also demonstrated an improvement in the clinical and immunological parameters in patients with MS after a 6-month intervention with EPO/HSO-enriched diet. The results showed significant improvements in the extended disability status score, as well as in the inflammatory status, as the pro-inflammatory cytokines (e.g., IFN-γ and IL-17) levels decreased and the anti-inflammatory cytokines IL-4) levels increased [101].

### 2.5. Coconut Oil

Coconut oil (CO) is obtained from coconut trees (Cocos nucifera), with a content of 65–75% of oil, and it has been used widely in food and industries [102]. Although CO may have some adverse effects because of its saturated fatty acid content [103], several biological activities of CO have been reported, including anti-oxidative and anti-inflammatory [104], as well as its ability to improve Alzheimer’s disease [105]. Extra virgin coconut oil (EVCO) has been suggested to be a nutritional alternative for patients with MS disease due to the ketone bodies obtained from EVCO, in addition to its numerous benefits linked to MS pathogenic mechanisms, including neuroprotective and anti-inflammatory effects [106]. Coconut oil was also reported to be a neuroprotective agent, showing a favorable effect on stroke incidence and survivability through histopathologic analysis of the brain using a stroke-prone spontaneously hypertensive rat model [107].

A recent human study showed that patients with MS were examined for a diet intervention enriched with EVCO for 4 months and supplemented with epigallocatechin gallate at 800 mg. The treated patients received 60 mL of extra EVCO divided into two equal intakes (30 mL in capsules for breakfast and 30 mL for lunch), and the control placebo received capsules containing microcrystalline cellulose, the same size and color, with EVCO for 4 months. The administration of EGCG and EVCO showed a neuroprotective effect, where a significant improvement in gait speed, quantitively balance and muscle strength were observed in the treated patients compared to the control patients, and this effect was due to the ketone bodies that may be formed from EVCO metabolism balance. This effect was suggested to be related to muscular improvements, which have been evidenced through the increase in ketone bodies in the blood [108].

Based on the literature, VCO contains saturated medium fatty acid that is readily absorbed in the gut [109], and the biotransformation of VCO into acetoacetate in the liver can be further metabolized into β-hydroxybutyrate (BHB) [110]. BHB is a by-product of lipid metabolism, which is known as a ketone body that has been reported to stand out for its neuroprotective effect observed after stroke and neurodegenerative diseases, as well as its anti-inflammatory effects [111]. Ketogenic diets have demonstrated neuroprotective, anti-inflammatory properties that may be effective for nondegenerative disorders including MS. Ketogenic diets have been shown to reduce ROS generation, upregulate antioxidant pathways, activate neuroprotective macrophages and suppress proinflammatory cytokine production [112,113]. Furthermore, the intervention of MS patients with a combination of EVCO with epigallocatechin gallate for 4 months has resulted in a significant decrease in the serum concentration of IL-6 and patients’ anxiety and an improvement in the functional capacity of the treated patients [114].

### 2.6. Walnut Oil

Walnut oil (WO) is obtained from walnut kernels (Juglans regia Linne) with a content of about 52–70% of oil, and it is a rich source of PUFA, accounting for 69–73%. Linolic acid is the major fatty acid, counting for 56%, and linolenic acid counts for 12%, while MUFAs account for 17.8–21.2% [115]. Walnut oil was noted to contain a considerable number of phenolic compounds. The main phenolic compound is tocopherol, and γ-tocopherol accounted for 80% of the total tocopherol. Walnut oil is also rich in phytosterols. B-sitosterol is the highest, accounting for 80% of the total phytosterol content [115]. Overall, WO has been reported to exert several health-benefitting activities, including cognitive impairment and memory deficits [116]. Moreover, WO has been shown to inhibit oxidative stress in the brain and prevent scopolamine-induced histological changes in hippocampal CA1 and CA3 neurons [117].

An experiment of the effect of WO on EAE animal model of MS was conducted by Ganji et al. [118]. Specifically, feeding mice a daily dose of 5 mL/kg of WO for 21 days showed a reduction in the severity of MS mice disorder and significantly decreased the serious sickness in the treated mice by reducing T-helper1 activity. Also, WO caused an improvement in the immune response, where it shifted from destructive to regulatory, suggesting that WO can be used in MS therapy. Walnut oil treatment enhances the T-helper 2 call response. It appeared that the reaction of T-helper 1 can be restrained by T-helper 2 cells through cytokine generation containing IL−4, IL-5, IL-10 and tumor growth factor-β. In mice treated with WO, there was a reduction in disorder severity and a modification in cytokine compared to mice not treated with WO. The possible mechanisms through which WO exerted its effect on reducing the seriousness of MS illness in EAE-treated mice were suggested to include anti-inflammatory mechanisms through the suppression of inflammatory cytokine production, the modulation of cytokine signal transduction pathways and the inhibition of adhesion molecule expression [118].

### 2.7. Essential Oil from Pterodon emarginatus Seeds

Essential oil from *Pterodon emarginatus* seeds (EOPS) is obtained from the seed of *Pterodon emarginatus*, which belongs to the Leguminosae family, and originates from Brazil. The chemical characterization of EOPS shows that the oil is composed of volatile aromatic terpenes including caryophyllene, β-elemene, germacrene-D, α-humulene, spathulenol and bicyclogermacrene [119]. Previous research reported that some of the natural triterpenes could modulate some of the immune response markers of EAE MS animal model experiments [120]. Essential oil from *Pterodon emarginatus* seeds had a positive effect in decreasing the development of autoimmune diseases by impairing both the B and T cell responses involved in disease development. The immunomodulatory effect of EOPS on collagen-induced arthritis animals has also been reported [121]. As a result, EOPS reduced the severity of arthritis and decreased the serum anti-CII IgG antibody and CD4 + CD69 + lymph node cell number compared to untreated animals.

Microglia is the bone marrow derived from resident macrophages of the central nervous system (CNS). Studies have used a localized activation of microglia as an in vitro model to study the pathogenesis of several neurodegenerative disorders, including Parkinson’s disease, Alzheimer’s disease and MS [122]. Alberti et al. [123] investigated the effects of EOPS on the progression of MS in vivo using an EAE model experiment and in vitro using microglia. EAE mice received oral treatment of E0PS at 50–100 mg/kg, while control animals were administrated with an oral Vehicle solution. The oral administration of EOPS at 100 mg/kg significantly reduced the neurological signs and development of MS in the EAE experiment compared to the untreated animals. The Th1 cell-mediated immune response was inhibited and the Treg response was upregulated by EOPS in the treated mice and microglial compared to the untreated animals and microglial cells. In addition, EOPS was able to inhibit the microglial activation and expression of iNOS synthase, associated with the inhibition of axonal demyelination and neuronal death, during the development of the disease. The inhibition of CD4+T lymphocytes, inhibition of microglial activation and reduction of the expression of pro-inflammatory mediators were the suggested mechanisms through which EOPS exerts its immunomodulatory effect in vivo and in vitro [123].

### 2.8. Flaxseed Oil

Flaxseed oil (FSO) is obtained from flaxseed (*Linum usitatissimum* L.), which is among the richest sources of α-linolenic, which counts for about 58%, followed by linoleic acid with 16% and oleic acid with 21% [124]. Flaxseed oil seems to have several health benefits, including anti-inflammatory [125] and antioxidant activities [126]. In addition, FSO was reported to improve cognitive function in healthy older adults and to improve verbal fluency performance [127]. Moreover, it was found that pretreatment with FSO exhibited neuroprotective effects on neurons of the motor cortex area and enhanced the functional motor recovery following cerebral I/R injury by increasing the brain-derived neurotrophic factor and glial cell-derived neurotrophic factor levels [128].

Regarding the effect of FSO on MS, only one study was found in the literature [129]. Jelinek et al. [129] conducted a study on a large cohort of MS patients. The information provided referred to the type of MS, disability health-related quality of life, relapse rates and frequency of fish consumption and ω-3 supplementations, mainly as flaxseed oil. Interestingly, a reduction in the relapse rate was seen at a large level (over 52%) for those MS patients who took flaxseed oil in univariate analysis. Also, FSO-supplemented MS patients were the strongest group, resulting in a significant reduction in disability. The authors claimed that FSO showed a stronger association with quality of life, disease activity and disability than fish oil. The possible mechanism of the FSO effect on MS might be due to its anti-inflammatory and antioxidant action [129]. As was shown, FSO supplementation into the diet of diabetic rats resulted in the enhanced activity and upregulation of the mRNA level of hepatic antioxidant enzymes and down-regulated the expression of hepatic inflammatory genes including TNF-α, IL-6, MCP-1, INF-γ and NF-κB. Therefore, the FSO diet prevented tissue injury and alleviated diabetes in diabetic rats [125]. 

### 2.9. Olive Oil

Olive oil is rich in MUFAs in the form of oleic acid, with a content of 55–83%, but also α-linolenic acid (3–19%), phenolic compounds, sterols, tocopherols, polar pigments (pheophytins and chlorophylls), triterpenic, dialcohols and hydrocarbons, including squalene and the carotene β-carotene and xanthophylls [130]. Olive oil was reported to inhibit food-borne pathogens and stimulate useful microorganisms like *L. acidophilus* and *B. bifidum*, which are known as probiotic strains, with potential health benefits after consumption. In addition, a diet rich in virgin olive oil (VOO) could modulate the gut microbiota in both animals and humans [131,132]. Studies have shown that olive oil has an anti-inflammatory effect in vivo and in vitro [133]. In addition, olive oil was reported to modulate the activation of pro-inflammatory genes and reduce inflammatory cytokine expression [134]. Moreover, there is accumulating evidence that the regular consumption of the Mediterranean diet, which contains VOO as a main ingredient, is associated with a reduction in developing chronic diseases such as cardiovascular diseases [135].

According to the literature, the natural antioxidants present in olive oil, including phenolic compounds and vitamin E, reduce neuron damage by inhibiting the generation of ROS, apoptosis, protein oxidation and damage to the cell membrane and by decreasing βamyloid toxicity [136]. Oleacein is a phenolic compound from extra virgin olive oil (EVOO), it is one of the main secoiridoids of the EVOO minor compounds that many health benefits of EVOO have been attributed to [137]. Furthermore, oleocanthal may have the ability to counter inflammation in the brain by decreasing the acting astrocytes activation and proinflammatory cytokines level [138]. Gutiérrez-Miranda et al. [139] examined the potential protective effect of olive oil secoiridoid oleacein on intestinal barrier dysfunction using the EAE female MS model. Olive oil was dissolved in normal saline containing 5% DMSO and mice were injected intraperitoneally with 10 mg/kg/day, while control mice were injected with a vehicle control solution of DMSO/saline for 24 days. Olive oil oleacein showed protection against EAE-induced superoxide an-ion and the accumulation of protein and lipid oxidation products in the colon. In addition, oleacein could enhance antioxidant activity. These effects reduced the colonic IL-1β and TNFα levels in the treated EAE mice. Olive oil leacein could effectively regulate intestinal oxidative stress, inflammation and permeability when administered to EAE mice. It was reported that the polyphenols present in EVOO reduced morbidity and slowed down the progression of neurodegenerative diseases [140]. In addition, EVOO polyphenols were also found to reduce inflammation and oxidative stress and modulate the immune system by affecting white blood cell activity and cytokine production [141]. According to Carito et al. [142], polyphenols of olive oil may have a role in the regulation of neurotrophic levels—and in particular, nerve growth factor (NGF) and brain-derived neurotrophic factor (BDNF)—in animal models and humans, and this effect was suggested through the potentiation of neurotrophins’ action [142]. 

Patients with MS can show cognitive and mental health impairment, which has been recognized as an important factor that affects the quality of life of MS patients [143]. Chatzikostopoulos et al. [144] studied the effects of early-harvest extra virgin olive oil (EH EVOO) on the cognition and mental health of primary or secondary progressive multiple sclerosis human patients. The multiple sclerosis patients were asked to consume three tablespoons of EH EVOO per day for one year. As a result, the consumption of EH EVOO for one year resulted in significant improvements in several MS symptoms including visuospatial memory and processing speed, and an improvement in functions related to the frontal lobes, such as mental flexibility and adaptation to the environment, when compared to untreated patients. In this line, EVOO was suggested to have an important role in neuroprotection as it prevents cognitive decline in humans [145]. The mediterranean diet, which is rich in EVOO, has been shown to prevent cognitive de-cline in Alzheimer’s disease in the elderly population [146].

Levels of fecal calprotectin is marker of short -term clinical outcome and presence of mucosal healing in patients with in inflammatory bowel disease [147]. A pilot study with relapsing-remitting multiple sclerosis (RRMS) patients, with 10 patients receiving treatment and 5 patients as the controls, was reported by Wozniak et al. [148]; it investigated the efficacy of high phenolic early harvest extra virgin olive oil on fecal calprotectin in patients with RRMS. The treated patients received 50 mL of EH-EVOO per day—25 mL of oil during the morning hours and 25 mL during the night hours for 4 months—while the control patients did not receive any oil. This study indicated a significant reduction in fecal calprotectin levels after 2 and 4 months of administration compared to patients that did not receive the oil. In addition, decreased inflammation was observed among the treated group. The reduction in inflammation through decreasing the fecal calprotectin was suggested to be due to the presence of polyphenols in the EH-EVOO [148].

**Figure 1:**
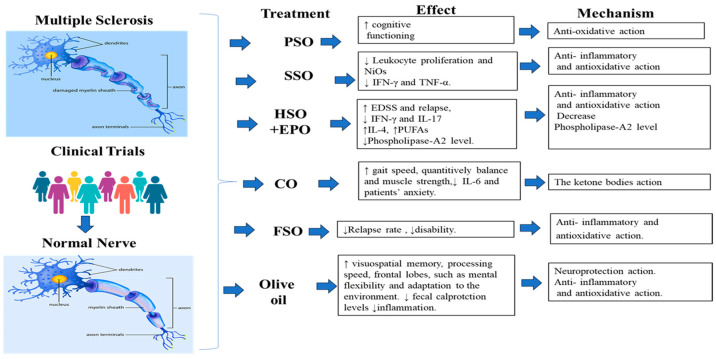
A summary of plant oils used in the treatment of MS in clinical trial studies. Image for Multiple Sclerosis and normal nerve was taken from https://www.santhoshselvam.com/blog/category/multiple-sclerosis/ (accessed on 5 June 2023) the third of June 2023. Clinical trial image was taken from https://mrctcenter.org/clinical-research-glossary/glossary-words/clinical-trial/ (accessed on the 5 June 2023).

**Figure 2:**
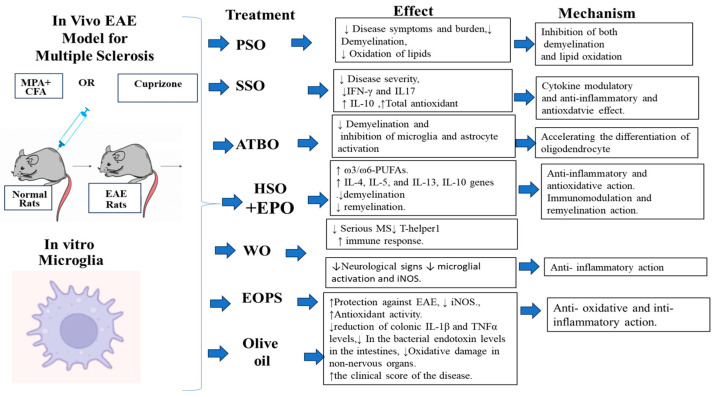
A summary of plant oils used in the treatment of MS in vivo EAE model and in vitro microglia studies.

Studies have evidenced the unbalanced oxidative status in different organs in patients with MS and EAE animal models including spinal cord, blood, serum, brain and cerebrospinal fluid [149,150]. In this regard, Conde et al. [151] reported the protective effect of EVOO and its compounds, including hydroxytirosol and oleic acid, on non-nervous organs, including the heart, kidney, liver and small and large intestines, that are affected by oxidative damage in an EAE model using rats. Treated rats received 10% of their calorie intake (in terms of weight) of EVOO, 2.5 mg/kg body weight of hydroxytirosol and had a gastric catheter. Consequently, the animals treated with EVOO showed a reduction in the bacterial endotoxin levels in the intestines and a reduction in oxidative damage in the non-nervous organs. Furthermore, the clinical score of the disease was improved in the treated animals compared to the untreated animals. The possible mechanism through which EVOO exerts its effect was related to the antioxidant and cytoprotective activity, including decreasing the lipopolysaccharide levels, which is related to inflammatory phenomena, and oxidative stress in the intestinal tissue and in other organs in the EAE model. A summary of the plant oils used in the treatment of MS is presented in Table 1 and Table 2 and Figure 1 and Figure 2.

## 3. Perspectives and Conclusions

The findings from the reviewed articles indicate that plant oils and their essential components, including MUFAs, antioxidants and polyphenols, could be effective in declining the progress of MS symptoms, primarily through reducing inflammation, immunomodulation and inhibition of oxidative stress and enhancing antioxidant activity. Nonetheless, the results from both animal and clinical trials are limited; most of the reviewed articles have many limitations as most of the studies were conducted with the use of a small sample size and were not replicated. Concerning the clinical trials, most of the studies were conducted with a low sample size and a short intervention period. There is no diversity in the analyzed articles. The papers found for each plant oil were from the same author group and country, and nearly half of the evaluated publications in this review are from Iran, particularly for HSO and EPO. In regard to one plant oil, there was no variety or changes in the selected concentrations of the oil or duration of the treatments that were used in either the clinical trial or EAE animals. Regarding the effect of both HSO and EPO, in the studies reported by Rezapour-Firouzi et al. [87,94,96,98], the same dose and time were used across the four publications. In addition, none of the studies mentioned the reason for the selected dosage and concentrations used for the treatments. Finally, the safety of the selected dose was not mentioned, with the exception of the study of PSO that was reported by Petrou et al. [45]. The evaluated human studies did not reveal whether the oils were accepted and consumed by the patients in an adherent manner, which is essential in order to be clear for the potential future goal of prescribing the use of oils to MS patients.

In regard to CO, the treatment was a combination of both CO and EGGG. There was no group that received only CO in order to clearly elucidate its effect independently. Most of the reviewed articles did not show detailed mechanisms of action. Regarding the placebo control, some studies used olive oil as a placebo control, as in the study of Rezapour-Firouzi et al. [96], while olive oil was reported to have a positive effect in reducing MS in vivo and in humans. In another study, soybean oil was used as a placebo control. As for ATBO, WO and EOPS, only EAE experiments were reported, and there was no clinical trial to support the EAE findings. 

An important documented scientific topic is the impact of plant oils and their essential constituents on human health. At present, biodiversity research is shifting away from the simple concept of conservation and preservation and is moving towards the utilization of natural resources in order to harness their characteristics and extend the concept of biodiversity toward functional biodiversity. Essential components such as MUFAs, antioxidants and polyphenols are naturally present in plant oils and could have an impact on MS through diet. As a lot of plant oils were found to be useful in slowing MS progression, their role in sustainable nutrition should be considered. With reference to plant oils, it is critical to look for plant oils with a higher content of these useful components and to explore the possibility of using them in MS treatment and prevention.

It is crucial to note that some of these plant oils are not widely accessible; therefore, it may be best to use the food source that the oil is derived from. For instance, pomegranate fruits and its peel are widely available and could be recommended for MS diseases. Stojanović et al. [152] reported that pomegranate peel extract was effective in reducing the level of Il-17 in animal models of MS and type 1 diabetes. This may interfere with MS expression, in addition to its ability to attenuate the activation of encephalitogenic T-cells. Such an event is fundamental in MS and encephalomyelitis disease development [1]. Additionally, the consumption of sesame seeds was shown to decrease human serum inflammatory indicators like IL-6, which may be advantageous for MS disease [153].

In conclusion, plant oils could potentially reduce MS progression. Larger studies with a longer duration are essential to confirm the effects of the reviewed plant oils. More plant oil types must be explored for their potential benefits on MS in order to be considered as suitable candidates for clinical and therapeutic usage in MS.

## Figures and Tables

**Table 1:** A summary of plant oil used in the treatment of MS clinical trial studies.

Plant Oil	Authors (Country)	Design of Study	Dosage	Duration of Study	Effects	Possible Mechanisms of Action and Principle Active Compounds
Pomegranate Seed Oil (PSO)	Petrou et al. [45] (Jerusalem)	Clinical trial with 30 MS patients, placebo pills containing soybean oil then additional PSO	Not mentioned	9 months	↑ cognitive functioning	Anti-oxidative action of PSO nanoemulsion and its main active ingredient punicic acid
Sesame seed oil (SSO)	Faraji et al. [62] (Iran)	Clinical trial, control treated with 30 μg/week.	30 μg/week of interferon beta-1a + 0.5 mL/kg/day of oral sesame oil	6 months	↓ Leukocyte proliferation and Nitric oxide ↓ IFN-γ and TNF-α	Anti- inflammatory and antioxidative action
Hemp seed oil and Evening primrose oil (HSO + EPO)	Majdinasab et al. [94] (Iran)	Clinical trial with 52 MS patients, with placebo Control		3 Months		Antioxidants and Inflammatory action.
	Rezapour-Firouzi et al., [96] (Iran)	Clinical trial, Placebo control of olive oil Each group has 20 patients.		6 Months	↑ Clinical improvements in expended disability status scale and relapse rate in MS Also, ↓ pro-inflammatory cytokines IL-17.	Antioxidant activity Inflammatory functions and cytokines production
	Rezapour-Firouzi et al., [97] (Iran)	Clinical trial, Placebo control of olive oil Each group has 23 patients.	1 g oral capsule containing EPO every 12 h for 3 months.	6 Months	↑ in red blood cells PUFAs and ↓ Phospholipase-A2 level.	Decrease Phospholipase-A2 level.
	Rezapour-Firouzi et al., [98] (Iran)	Clinical trial, Placebo control of olive oil Each group has 23 patients.	A combination of HSO and EPO with 9/1 ratio AT 18–21 g/day (6–7 g, three times daily)	6 Months	↑ extended disability status score.	
	Rezapour-Firouzi et al., [87] (Iran)	Clinical trial, Placebo control of olive oil Each group has 20 patients.	A combination of HSO and EPO with 9/1 ratio AT 18–21 g/day (6–7 g, three times daily)	6 Months	↓ Pro-inflammatory cytokines of IFN-γ and IL-17 and ↑IL-4.	↓ Pro-inflammatory and ↑ in anti-inflammatory cytokines.
Coconut oil (CO)	Cuerda-Ballester et al., [109] (Spain)	Clinical trial of 27 MS patients, Placebo Control of 26 patients.	60 mL of extra virgin CO supplemented with EGCG.	4-month	↑ improvement in gait speed, quantitively balance and muscle strength.	The ketone bodies that may formed from EVCO metabolism balance.
	Platero et al., [115] (Spain)	Clinical trial of 24 MS patients and with Placebo Control of 27 patients.	60 mL of extra virgin coconut oil supplemented with epigallocatechin gallate (EGCG)at 800 mg.	4 Months	↓ in serum concentration of IL-6 and patients’ anxiety. ↑ Improvement in patients’ functionality.	The ketone bodies that may formed from CO metabolism balance.
Flaxseed oil (FSO)	Jelinek et al., [130] (Australia)	Surveyed study with MS patients through Web 2.0 platforms, including social media.	last 12 months.	A large cohort of 2469 people with MS disease	↓ Relapse rate was seen at large level (over 52%) for those MS patients who are taking FSO in univariate analysis. ↓ of disability.	anti-inflammatory and antioxidant action of PUFAs
Olive oil	Chatzikostopoulos et al. [146] (Greece)	Clinical trials With 30 MS patient 20 for intervention and 10 controls	Three tablespoons of EVOO/day	6 Months to one year	↑ Improvement visuospatial memory, processing speed and improvement in functions related to the frontal lobes, such as mental flexibility and adaptation to the environment when compared to control patients.	Neuroprotection action.
	Greta Wozniak et al. [150] (Cyprus)	Clinical trials with RRMS patients 10 patients for treatment and 5 patients as control.	50 mL of EVOO/day, Control patients did not take any oil.	2–4 months	↓ Reduction in fecal calprotectin levels after 2 and 4 months of administration, ↓ inflammation in the treated group.	↓ Inflammation by decreasing the fecal calprotectin due to the presence of t polyphenols in the oil.

**Table 2:** A summary of plant oils used in the treatment of MS, in vivo EAE model and in vitro microglia studies.

Plant Oil	Author (Country) Reference Number	Design of Study	Dosage	Duration of Study	Effects	Possible Mechanisms of Action and Principle Active Compounds
Pomegranate Seed Oil (PSO)	Binyamin et al. [52] (Jerusalem)	EAE model of MS in female mice	PSO as nanoemulsion by gavage and PSO with diet at 25 or 75 mL/kg of the diet.	10 days	↓ Disease symptoms and burden, ↓ Demyelination, ↓ Oxidation of lipids in the brains of EAE mice.	Inhibition of both demyelination and lipid oxidation
Sesame seed oil (SSO)	Javan et al., [68] (Iran)	EAE model of MS in female mice	4 mL/kg/day) injected intraperitoneally. Control was injected with 4 mL phosphate buffer intraperitoneally.	20 days	↓ Disease severity, ↓ IFN-γ and IL17 ↑ IL-10	Cytokine modulatory and anti-inflammatory effect.
	Mosayebi, et al., [71] (Iran)	EAE model of MS in male mice	4 mL/kg/day) injected intraperitoneally (10 mice).	25 days	↓ Clinical symptoms of EAE, ↑ Total antioxidant capacity.	Inhibition of oxidative stress
Acer truncatum Bunge seed oil (ATBO)	Xue et al., [77] (China)	Cuprizone induced mice as a MS model	4% ATBO of the diet.	2 Weeks	↓ Demyelination and inhibition of microglia and astrocyte activation in vitro.	Accelerating the differentiation of oligodendrocyte precursor cells to mature oligodendrocytes in vitro.
Hemp seed oil and Evening primrose oil (HSO + EPO)	Rezapour -Firouzi et al., [89] (Iran)	EAE model of MS in female mice	Oral EPO/HSO (50 λ/mouse)	2 weeks	↑ The percentage of essential fatty acids and ω3/ω6-PUFAs. ↑ The expression levels of IL-4, IL-5 and IL-13 genes No demyelination in the brain and spinal cord sections of the EPO/HSO treated mice.	Antioxidants and PUFAs presented in both oils are the responsible compounds for the effect through anti-inflammatory and antioxidative action.
	Rezapour-Firouzi et al., [92] (Iran)	EAE model of MS in female mice	A combination of HSO and EPO at λ/mouse	28 days after	↓ of MS diseases in EAE. ↑ the expression of IL-10 gene, ↓ cell infiltration and promote remyelination.	Immunomodulation and remyelination activities.
Walnut oil (WO)	Ganji et al., [118] (Iran)	EAE model of MS in female mice	Gavage daily with 5 mL of WO/kg b.w phosphate buffered saline.	21 days	↓ Serious MS Sickness, ↓ T-helper1 activity and ↑ improvement of immune response	Anti-inflammatory mechanisms
Essential oil from *Pterodon emarginatus* seeds (EOPS)	Alberti et al., [124] (Brazil)	EAE model of MS in mice and in vitro using microglia macrophages cells of the central nervous system	Oral treatment of E0PS dissolved in Tween 80 and 0.9% NaCl at 50–100 mg/kg.	25 days	↓ Neurological signs and the development of MS diseases in EAE animals ↓ microglial activation and expression of iNOS.	Inhibition of microglial activation and reduce the expression of pro-inflammatory mediators and reduce the oxidative stress.
Olive Oil	Gutiérrez-Miranda et al., [141] (Greece)	EAE model of MS in female mice	Treated mice with olive oil were injected intraperitoneally With 10 mg/kg/day	24 days	↑Protection against EAE ↓ superoxide anion and lipid oxidation products in colon, ↑ Antioxidant activity. ↓ reduction in colonic IL-1 β and TNFα levels.	Anti-oxidative and inti-inflammatory mechanisms.
	Conde et al. [151] (Spain)	EAE model of MS in rats	10% of the calorie intake (in terms of weight) is EVOO with a gastric catheter.	51 days	↓ In the bacterial endotoxin levels in the intestines, ↓ Oxidative damage in non-nervous organs. ↑ the clinical score of the disease.	Anti-oxidative damage. in the EAE model
